# Wirkungen digitaler Medien auf die Gesundheit von Kindern und Jugendlichen mit Schwerpunkt auf dem Verzehr ungesunder Lebensmittel

**DOI:** 10.1007/s00103-024-03834-4

**Published:** 2024-01-17

**Authors:** Antje Hebestreit, Elida Sina

**Affiliations:** Abteilung: Epidemiologische Methoden und Ursachenforschung, Leibniz-Institut für Präventionsforschung und Epidemiologie – BIPS, Achterstr. 30, 28359 Bremen, Deutschland

**Keywords:** Medienzeit, Medien-Multitasking, Kognitive Flexibilität, Lebensmittelmarketing, Metabolisches Syndrom, Media time, Media multitasking, Cognitive flexibility, Food marketing, Metabolic syndrome

## Abstract

Die Medienzeiten von Kindern und Jugendlichen haben seit 2019 zugenommen. Längere Zeiten, die z. B. mit Medien-Multitasking verbracht werden, werden zunehmend als Einflussfaktor auf die Gesundheit diskutiert. In dieser narrativen Übersichtsarbeit wird die Bedeutung der Nutzungsdauer für die Entstehung von Adipositas und metabolischen Gesundheitsendpunkten bei Kindern und Jugendlichen beleuchtet und Zusammenhänge mit dem Verzehr von ungesunden Lebensmitteln, z. B. durch eine erhöhte Exposition gegenüber Medien-Lebensmittelmarketing, vertiefend besprochen.

Lange Nutzungszeiten scheinen positiv mit Geschmackspräferenzen für süß, fettig und salzig sowie mit einer erhöhten Snack- und Energiezufuhr assoziiert zu sein. Langfristig stehen zunehmende Medienzeiten überdies mit einem erhöhten Risiko für das metabolische Syndrom und seine Einzelkomponenten in Beziehung. Ein besonderer Nutzen sozialer Medien für die Förderung von körperlicher Aktivität und gesunder Ernährung liegt in der erleichterten Einbeziehung sonst schwer erreichbarer Gruppen. Soziale Medien können ferner die soziale Unterstützung durch Gleichaltrige vereinfachen und so das Wohlbefinden junger Nutzer:innen positiv beeinflussen.

Insbesondere vor dem Hintergrund der noch nicht vollständig entwickelten kognitiven Fähigkeiten von Kindern und Jugendlichen werden im Artikel Handlungsoptionen zum Schutz junger Nutzer:innen angesprochen. Die Exposition gegenüber bestimmten Medieninhalten scheint negative Auswirkungen auf die Nahrungsmittelauswahl und das Essverhalten von jungen Nutzer:innen zu haben. Dadurch wird der Appell gestützt, digitale Werbung für Lebensmittel und Getränke, die sich an Kinder und Jugendliche richtet und die nicht den WHO-Kriterien für kindergerechte Lebensmittel entspricht, in diesen Medien stark einzuschränken.

## Abstract (en)

Children and adolescents’ media time has increased since 2019. Prolonged time spent with media and media multitasking is increasingly being discussed as a health determinant. This narrative review highlights the importance of media time on the development of obesity and metabolic disorders in children and adolescents and discusses in more detail the associations with consumption of unhealthy foods and increased exposure to media food marketing.

The prolonged durations that children and adolescents spend with social and digital media in their leisure time are positively related to sensory taste preferences for sweet, fatty, and salty foods as well as to higher snack food and energy intakes. Moreover, prolonged media time is also associated with a long-term increased risk of metabolic syndrome and its single components. The potential of social media for promoting physical activity and healthy eating lies especially in simplifying the inclusion of otherwise hard-to-reach groups. Further, social media may facilitate social support from peers and thus positively influence the well-being of young users.

Particularly against the background of the not yet fully developed cognitive abilities of children and adolescents, the article refers to options for action to protect young media users. Exposure to certain social media content appears to negatively relate to children and adolescents’ food choices and eating behaviors, supporting the call to regulate advertising directed at young consumers for foods and beverages that do not meet WHO criteria for child-friendly foods in these media.

## Hintergrund

Kinder und Jugendliche wachsen heute in einer Umgebung auf, in der digitale Medien allgegenwärtig und rund um die Uhr verfügbar sind: In Deutschland besitzen 96 % der 12- bis 19-Jährigen ein eigenes Smartphone, 73 % einen eigenen Computer oder Laptop. Mehr als die Hälfte der Kinder- und Jugendzimmer sind mit Fernsehgeräten, einem Smart-TV oder einer Spielekonsole ausgestattet; der Zugang zu den Lieblingsinternetseiten, Streamingdiensten (Musik und Filme) sowie digitalen Spielen ist in der Freizeit somit jederzeit möglich. Kinder und Jugendliche nutzen diese Medien zum Lernen und zur Selbstentwicklung, für die Freizeitgestaltung und Unterhaltung, zur Kommunikation mit Gleichaltrigen und der Familie sowie zum Empfangen oder Teilen von Inhalten über soziale Medien und ihre Plattformen [1].

In Deutschland sind die Medienzeiten junger Menschen seit 2019 gestiegen. Kinder zwischen 6 und 13 Jahren nutzen täglich ca. 240 min ein oder mehrere Geräte; Jugendliche zwischen 12 und 19 Jahren sind im Schnitt 224 min online [1, 2]. Damit übersteigen die Nutzungszeiten die von der Bundeszentrale für gesundheitliche Aufklärung (BZgA) formulierten Orientierungswerte für diese Bevölkerungsgruppen: Kinder zwischen 3 und 6 Jahren sollten täglich höchstens 30 min Bildschirmmedien nutzen und Kinder bis 10 Jahre täglich höchstens 45–60 min. Eltern von Kindern ab 12 Jahren sollen Bildschirmzeiten „nach Augenmaß“ begrenzen [3]. Die Weltgesundheitsorganisation (WHO) empfiehlt insbesondere die Medienzeit in der Freizeit zu begrenzen, d. h. in der Zeit, die nicht für Schule, Hausaufgaben oder Studium aufgewendet wird [4].

Die während der SARS-CoV-2-Pandemie gestiegenen Medienzeiten von Kindern und Jugendlichen bleiben trotz der Aufhebung öffentlicher Beschränkungen auch nach der Pandemie hoch. Weitere Untersuchungen sind aber erforderlich, um festzustellen, ob der Trend auf das frühere Niveau zurückgeht. Während der SARS-CoV-2-Pandemie stieg die freizeitbezogene Medienzeit stärker an als die Medienzeit für Bildung [5], was darauf hindeutet, dass die Digitalisierung in den Schulen und das E‑Learning möglicherweise nicht die Hauptursachen für diesen Anstieg sind. Darüber hinaus wurde berichtet, dass 11,2 % der Kinder in der zweiten Pandemiewelle ein *Social-Media*-Konto hatten, verglichen mit 4,4 % vor der Pandemie [6]. Auch bei Kindern im Vereinigten Königreich nahm die *Social-Media-*Exposition während der SARS-CoV-2-Pandemie zu, mit einem größeren Anstieg bei Mädchen und bei Kindern aus Familien mit niedrigerem Bildungsniveau [7].

Lange Medienzeiten von Kindern und Jugendlichen, insbesondere bei der gleichzeitigen Nutzung verschiedener Medien, werden zunehmend als Einflussfaktor auf die Gesundheit diskutiert, weil sie Auswirkungen auf kognitive Kontrollprozesse haben können [8], welche wiederum mit ungesunden Verhaltensweisen und einem höheren Adipositasrisiko in Zusammenhang stehen [9].

Ziel dieses narrativen Übersichtsartikels ist, die Relevanz der Nutzungsdauer für die Entstehung des metabolischen Syndroms und seiner Einzelkomponenten, wie Adipositas, bei Kindern und Jugendlichen zu beleuchten. Welche Rolle der Lebensmittelverzehr in diesem Zusammenhang spielt, wird erklärend besprochen. Insbesondere auf die Exposition gegenüber Medien-Lebensmittelmarketing wird vor dem Hintergrund der noch nicht vollständig entwickelten kognitiven Fähigkeiten von Kindern und Jugendlichen eingegangen. Zusammenhänge mit einer verringerten körperlichen Aktivität und verkürzten Schlafzeiten als weitere Determinanten von Gesundheit werden kurz ergänzt. Abgeschlossen wird der Artikel mit Erkenntnissen zu gesundheitsfördernden Aspekten digitaler Medien und einer kurzen Betrachtung der Rahmenbedingungen.

Der Artikel fasst aktuelle Erkenntnisse aus der Identification and prevention of Dietary- and lifestyle-induced health EFfects In Children and infantS (IDEFICS)/ I.Family-Kohortenstudie sowie einer eigenen systematischen Übersichtsarbeit zusammen [10–13] und ergänzt die Ergebnisse mit aktueller Literatur zum Thema. Der Artikel möchte den aktuellen Wissensstand zum Thema zusammenfassen und erhebt keinen Anspruch auf Vollständigkeit. Die IDEFICS/I.Family-Kohorte ist eine multizentrische Kinder- und Jugendkohorte in 9 europäischen Ländern mit 4 Messzeitpunkten [14]. Die IDEFICS-Studie untersuchte von 2006 bis 2010 die Rolle gesundheitsbezogener Verhaltensweisen und ihrer Determinanten bei der Entwicklung von Fettleibigkeit und Stoffwechselstörungen. Insgesamt 16.230 Kinder im Alter von 2 bis 9 Jahren nahmen an der Basiserhebung (2007–2008) teil. Davon wurden 13.587 Kinder während der ersten Folgeerhebung (2009–2010) erneut untersucht. An der zweiten Folgeerhebung (2013–2014), die im Rahmen der I.Family-Studie stattfand, nahmen 7123 Indexkinder (7 bis 17 Jahre) erneut teil [14]. Die aktuellste Folgeerhebung wurde 2020–2021 mit 5100 Teilnehmenden als Online-Survey durchgeführt.

## Digitale Medienzeiten und kardiometabolische Gesundheit

Immer mehr Studien berichten, dass die Gesamtzahl der Stunden, die Kinder und Jugendliche täglich mit digitalen Medien verbringen, mit dem Alter zunimmt [15, 16]. Frühere Studien zu Medienzeiten und dem metabolischen Syndrom bei Kindern und Jugendlichen fanden nicht immer überzeugende Belege für einen Zusammenhang [17, 18]. Daher untersuchten Sina et al. [12] basierend auf Daten der IDEFICS/I.Family-Kohorte mögliche Zusammenhänge für individuelle altersabhängige Verläufe von Medienzeiten, die als zufällige Abschnitte (Stunden/Tag) und als lineare Steigungen (Stunden/Tag/Jahr) dargestellt wurden. Ferner wurde die Abweichung von der mittleren Medienzeit in der Kindheit (2–16 Jahre) berechnet. Die Medienzeiten sind Gesamtzeiten, die in der Freizeit mit Fernsehen, PC, Spielekonsole und Internet verbracht wurden. Die Analysen nutzten die von Ahrens et al. [19] entwickelte Definition für das metabolische Syndrom im Kindesalter sowie seine Einzelkomponenten abdominale Adipositas, Dyslipidämie, Insulinresistenz und Bluthochdruck.

Die selbstberichteten Nutzungszeiten stiegen mit zunehmendem Alter der Studienteilnehmenden an: Verbrachten 2‑Jährige in ihrer Freizeit im Mittel täglich 2,2 h mit digitalen Medien, nutzten 16-Jährige digitale Medien täglich 4,2 h. Die Nutzungsverläufe waren positiv mit dem metabolischen Syndrom und seinen Einzelkomponenten assoziiert: Kinder mit steigenden Verläufen von Medienzeiten über dem Mittelwert hatten ein 30 % höheres Risiko, ein metabolisches Syndrom zu entwickeln. Diese Trends waren unabhängig vom Bildungstand der Eltern. Bei Jungen war der Anstieg der Medienzeiten steiler und das Risiko für ein metabolisches Syndrom höher als bei Mädchen [12].

Die Ergebnisse bestätigen die Beobachtungen früherer Studien; diese zeigten ein doppelt so hohes Risiko für ein metabolisches Syndrom für Jugendliche mit Medienzeiten ab 2 h täglich [20]. Das höchste Risiko für ein metabolisches Syndrom wurde ab einer Bildschirmzeit von täglich 5–6 h berichtet [20]. Auch das Risiko für Übergewicht stieg ab einer Medienzeit von 2 h bei Jugendlichen [21] und ab 60 min bei unter 10-Jährigen [22].

Als mögliche Gründe für diese Beobachtungen werden u. a. übermäßiges Sitzen und Bewegungsmangel sowie kürzere Schlafzeiten diskutiert. Letztere werden der Vollständigkeit halber im Folgenden kurz angesprochen. Untersuchungen bei Kindern und Jugendlichen zeigten, dass im Vergleich zu anderen Verhaltensweisen Zeit, die mit Sitzen oder in einem geringen Aktivitätsniveau verbracht wurde, positiv mit Risikomarkern für Adipositas assoziiert war. Hingegen steht die Zeit, die in moderater bis starker körperlicher Aktivität verbracht wurde, in bidirektionalem Zusammenhang mit dem Gewichtsstatus bei Kindern und Jugendlichen [23].

Smartphones und Tablets ermöglichen eine Gerätenutzung im Schlafzimmer und erschweren im Vergleich zu klassischen Fernsehern besonders zur Schlafenszeit die Kontrolle durch die Eltern. Das blaue und helle Licht, das von diesen digitalen Geräten ausgestrahlt wird, scheint auf den zirkadianen Rhythmus zu wirken, das Einschlafen zu verzögern und die Gesamtschlafdauer zu verkürzen [24]. Kinder und Jugendliche mit längeren Bildschirmzeiten berichteten eine spätere Einschlafzeit und kürzere Schlafzeiten [25], wobei davon ausgegangen wird, dass eine interaktive Bildschirmzeit wie bei der Computernutzung den Schlaf stärker beeinträchtigt als eine passive Bildschirmzeit wie beim Fernsehen [26]. Sowohl spätere Einschlafzeit als auch verkürzte Schlafzeiten sind mit ungesunden Verhaltensweisen in dieser Altersgruppe assoziiert, wie z. B. dem häufigen Verzehr von Snacks und Fast Food, dem seltenen Verzehr von gesunden Lebensmitteln, zu wenig körperliche Aktivität und längere Sitzzeiten, was langfristig das Risiko für Übergewicht und für kardiometabolische Erkrankungen erhöhen kann [27–29].

## Digitale Medienzeit, Lebensmittelmarketing und das Ernährungsverhalten

Neben den o. g. Verhaltensweisen werden auch ungesunde Ernährungsgewohnheiten mit längeren Bildschirmzeiten in Verbindung gebracht, wie z. B. die Neigung, häufiger fett- und zuckerhaltige Lebensmittel zu verzehren [22], die langfristig die Entwicklung von abdominaler Fettleibigkeit begünstigen kann [30]. Einer der wichtigsten Faktoren für die Auswahl und den Verzehr von Lebensmitteln ist die Geschmackspräferenz [31]. In der I.Family-Studie wurde, basierend auf selbstberichteten Angaben zu Lebensmittel- und Getränkepräferenzen, der Zusammenhang zwischen Medienzeiten und Geschmackspräferenzen für süß, fett, bitter und salzig von Kindern und Jugendlichen untersucht. Die Kinder und Jugendlichen verbrachten täglich durchschnittlich 2,4 h in ihrer Freizeit mit Fernsehen, dem Computer/der Spielekonsole, dem Smartphone oder dem Internet. Längere digitale Medienzeiten waren positiv mit Geschmackspräferenzen für süß, fettig und salzig, aber negativ mit der Präferenz für bitter assoziiert. Bitter ist ein Indikator für eher gesunde Lebensmittel wie Vollkornprodukte, Obst und Gemüse [10].

Eine Erklärung für diese Ergebnisse kann das Lebensmittelmarketing über Werbespots und über Influencer:innen bieten [32]. Kinder und Jugendliche sehen täglich in den digitalen Medien mehr als 15 Werbungen für ungesunde Lebensmittel, 92 % davon sind für Produkte, die nicht den WHO-Kriterien für kindergerechte Lebensmittel entsprechen [33]. Ferner kann das Essen vor dem Bildschirm vom Sättigungsgefühl ablenken und das Anschauen von Lebensmittelwerbung die Lust auf diese beworbenen oft hochverarbeiteten Lebensmittel erhöhen [32].

Die Industrie macht sich das Wissen zur Reaktionsfähigkeit des Gehirns bei der Entwicklung von Werbespots zunutze [34]. Das Gehirn von Kindern und Jugendlichen reagiert beim Anblick von appetitlichen Lebensmitteln stärker als das Gehirn Erwachsener: Untersuchungen mittels funktioneller Magnetresonanztomographie zeigten, dass Gehirnregionen, in denen Belohnung, Motivation und Erinnerung verarbeitet werden, beim Anblick von ungesunden Lebensmitteln stärker aktiviert wurden als beim Anblick von gesunden Lebensmitteln [35]. Außerdem fiel es Kindern und Jugendlichen schwerer, Essentscheidungen zu treffen, die ihren Vorlieben widersprachen, und z. B. gesündere Lebensmittel zu wählen, die ihnen weniger gut schmeckten [36]. Kinder scheinen also empfänglich für die ungesunden Lebensmittelverlockungen zu sein, die sie umgeben, und sie können die langfristigen Konsequenzen für ihre Gesundheit noch nicht abschätzen [37].

Die eigene systematische Übersichtsarbeit betrachtet insbesondere jene Mechanismen, die Zusammenhänge zwischen Social-Media-Inhalten und der Ernährung von Kindern und Jugendlichen erklären [13]. Relevante Datenbanken wurden nach Studien durchsucht, die den Zusammenhang zwischen sozialen Medieninhalten und Nahrungsmittelpräferenz sowie Ernährungsverhalten bei gesunden Kindern und Jugendlichen im Alter von 2–18 Jahren untersuchen. Es zeigte sich, dass Nutzer:innen nach dem Betrachten von Online-Bildern und Videos täglich mehr Süßigkeiten, frittierte Lebensmittel und zuckergesüßte Getränke, aber weniger Obst und Gemüse verzehren, unabhängig vom Alter. Das Betrachten von Bildern mit ungesunden Lebensmitteln aktivierte Gehirnregionen, die mit Erinnerung, Belohnung, Aufmerksamkeit und Entscheidungsfindung zusammenhängen, stärker als das Betrachten von Bildern mit gesunden Lebensmitteln oder ohne Lebensmittel. Kinder und Jugendliche verarbeiteten Lebensmittelwerbung aus den sozialen Medien unterschiedlich: Je bekannter die Influencer:innen waren, umso besser erinnerten sich Kinder und Jugendliche später an das beworbene Lebensmittel. Die Portionsgröße und die Energiedichte der Lebensmittel auf den Bildern, aber auch der Appetitzustand beim Betrachten spielten eine Rolle bei der Verarbeitung der Bilder und bei der anschließenden Nahrungsaufnahme.

Es wurden keine Belege für die Auswirkungen von sozialen und digitalen Medien auf die Verbesserung der Ernährungsqualität und der Ernährungskompetenz von Kindern und Jugendlichen gefunden [32, 38–40]. Zwar ist limitierend zu bedenken, dass die Ergebnisse auf selbstberichten Angaben und auf Querschnittsdaten basierten [38, 39], dennoch stimmen die Ergebnisse mit der Mehrheit der randomisierten kontrollierten Studien überein, die keinen Zusammenhang mit einer gesundheitsfördernden Ernährungsweise junger Nutzer:innen fanden [32, 38, 40]. Eine Erklärung könnte sein, dass die Präsentation ungesunder Lebensmittel als gesundheitlich positiv verstanden wird und im Zusammenhang mit unterhaltsamen Medieninhalten eine spätere Reflexion über die gesunden oder ungesunden Eigenschaften dieser Lebensmittel erschwert [41].

Die Ergebnisse legen nahe, dass Influencer:innen erfolgreich die Lebensmittelpräferenzen und -auswahl von Kindern und Jugendlichen beeinflussen können, indem sie mittels Empfehlungsmarketings Lebensmittel in ihren sozialen Medien anpreisen [42].

## Digitale Medien und kognitive Funktionen

Funktionelle und strukturelle Veränderungen des kindlichen Gehirns werden durch Interaktionen mit der Umgebung geformt [43]. Die Literatur liefert Hinweise darauf, dass die Exposition gegenüber digitalen Medien, einschließlich Smartphones, Internet und Medien-Multitasking, ein unabhängiger Risikofaktor für die kognitive Leistungsfähigkeit von Kindern und Jugendlichen ist [44]. Ebenso deuten Forschungsergebnisse darauf hin, dass lange Medienzeiten das Risiko für emotionale Probleme und geringes Wohlbefinden von Grundschüler:innen erhöhen [45] und dass ein geringes Wohlbefinden wiederum emotionsgetriebene Impulsivität verstärkt [46].

Durch die ständige Verfügbarkeit von mobilen Geräten und Internetzugang verbringen Kinder und Jugendliche immer mehr Zeit mit der Nutzung mehrerer Mediengeräte gleichzeitig (Medien-Multitasking; [47]). Untersuchungen mit 8‑ bis 12-Jährigen zeigten, dass auch die Anzahl der gleichzeitig genutzten digitalen Medien während des Medien-Multitaskings mit dem Alter zunimmt [15]. Die Fähigkeit zum Multitasking ist mit entwicklungsbedingten Veränderungen während der Kindheit und Jugendzeit verbunden [48]. In diesem Altersabschnitt besteht auch das Potenzial für eine beachtliche Neuroplastizität in jenen Gehirnregionen, die an der kognitiven Kontrolle beteiligt sind [49]. Ein hohes Maß an Medien-Multitasking wurde sowohl mit einer verbesserten inhibitorischen Kontrolle bei Jugendlichen in Verbindung gebracht [8] als auch mit einer herabgesetzten Impulskontrolle [9]. Dieses erklärt sich durch ein Ungleichgewicht zwischen den an der Selbstregulierung und der Belohnung beteiligten Gehirnregionen [9]. Die erhöhte Sensibilität für Außenreize bei jungen Erwachsenen mit langen Medien-Multitaskingzeiten wurde mit einer übermäßigen Nahrungs- und Energiezufuhr assoziiert und kann langfristig das Übergewichtsrisiko erhöhen [9].

Einschränkend ist zu erwähnen, dass für Kinder und Jugendliche überwiegend Daten aus Laborstudien verfügbar sind [50]. Studien mit multivariaten Datensätzen zur Art und Weise der Mediennutzung sind oft recht klein [15]. Für Kinder und Jugendliche sind großangelegte populationsbasierte Studien mit einer tiefen Phänotypisierung selten. Die I.Family-Studie füllt diese Datenlücke und eignet sich daher besonders, Medien-Multitasking mit den täglichen Zeiten der gängigsten Medienarten von Kindern und Jugendlichen (8–18 Jahre) zu beschreiben, z. B. Internet, Smartphone, Computer/Spielekonsole und Fernsehen. Mithilfe des Klassifikationsverfahrens der latenten Klassenanalyse wurden in der I.Family-Studie 4 Medien-Multitasking-Nutzungsmuster identifiziert. Sie ermöglichen es, die Auswirkungen der häufigsten Medienaktivitäten und des Multitaskings mit diesen Medientypen auf die exekutiven Funktionen zu untersuchen: kognitive Inflexibilität (fortgesetzte Fehlreaktionen trotz Änderung von Umweltregeln), Entscheidungsfähigkeit (Auslösen einer zielbezogenen Handlung) und emotionsgetriebene Impulsivität (durch negative Emotionen gesteuerte impulsive Reaktionen).

Es zeigte sich, dass jede zusätzliche Stunde, in der ein Smartphone genutzt wurde und eine zusätzliche Medienaktivität stattfand, die kognitive Inflexibilität und emotionsgetriebene Impulsivität bei Kindern und Jugendlichen verstärkte. Zusätzlich sank die Entscheidungsfähigkeit der jugendlichen Nutzer:innen. Hinsichtlich der 4 identifizierten digitalen Mediennutzungsmuster zeigten Kinder und Jugendliche mit hoher Smartphone‑/Internetnutzung, mittlerer TV- und geringer PC-Nutzung höhere Werte für kognitive Inflexibilität und Impulsivität, aber niedrigere Werte für die Entscheidungsfähigkeit im Vergleich zu Jugendlichen mit kurzen digitalen Medienzeiten [11].

Die Ergebnisse unterstützen frühere Beobachtungen, bei denen junge Erwachsene mit langen Medien-Multitaskingzeiten möglicherweise eine Veränderung des Gleichgewichts zwischen Kontroll- und Belohnungsregionen aufweisen und dadurch besonders empfindlich auf äußere Lebensmittelreize reagierten [9]. Auch Analysen der I.Family-Studie legten Zusammenhänge zwischen einer hohen emotionsgetriebenen Impulsivität, einer geringen kognitiven Flexibilität und herabgesetzten Entscheidungsfähigkeit mit dem Verzehr energiereicher Snacks im Kinder- und Jugendalter offen [37]. Diese Beobachtungen lassen sich mit der Bedeutung der kognitiven Fähigkeiten für das Treffen gesunder Entscheidungen erklären, die als „Neuroselektion“ bezeichnet werden [51]. Bei Kindern mit beeinträchtigten kognitiven Fähigkeiten ist es wahrscheinlicher, dass sie ungesündere Entscheidungen treffen und ein ungesünderes Verhalten aufweisen [51]. Einschränkend ist zu erwähnen, dass wegen des Querschnittscharakters der I.Family-Studie Schlussfolgerungen über kausale Zusammenhänge nicht getroffen werden können. Es kann ebenso davon ausgegangen werden, dass frühere Verzögerungen in der Entwicklung der kognitiven Funktionen zu längeren Medienzeiten bei Kindern und Jugendlichen geführt haben.

Es gibt Studien, die diese Ergebnisse nicht stützen [52, 53], insbesondere sind dies jene, die selbstberichte Exekutiv- und Aufmerksamkeitsfunktionen vor allem in jungem Alter auswerteten [8, 54, 55]. Ferner scheint die Nutzung von Videospielen bei Kindern mit entwicklungsbedingten Koordinationsstörungen kurzfristig positive Auswirkungen auf die kognitiven Funktionen zu haben. Es sind jedoch noch weitere Untersuchungen erforderlich, um die Nachhaltigkeit der Effekte bewerten zu können [56].

## Förderliche Aspekte digitaler Medien

Trotz allem bietet die digitale Umgebung Kindern und Jugendlichen vielfältige Möglichkeiten zum Lernen, zur Selbstentfaltung und zur Unterhaltung. Bei Jugendlichen wurden Zusammenhänge zwischen Inhalten sozialer Plattformen und der Identitätsentwicklung, dem Engagement und der sozialen Unterstützung durch Gleichaltrige sowie einer positiven Selbstdarstellung beobachtet; alle Aspekte sind der gesunden Entwicklung Heranwachsender förderlich. Soziale Medien erleichtern es Jugendlichen, von anderen zu lernen, wie sie mit schwierigen Situationen umgehen. Darüber hinaus ermutigen moderierte Diskussionsforen zu offenen Gesprächen über schwierige Themen, wodurch die Isolation verringert und die Bewältigung mentaler Probleme unterstützt wird [57]. Digitale Medien dienen jungen Menschen zur Aufnahme und Pflege von sozialen Kontakten und auch dazu, ihre Ansichten und Meinungen zu Themen, die sie interessieren, mit Gleichgesinnten zu teilen oder auf dem Laufenden zu bleiben. Sie erleichtern ihnen den Zugang zu Informationen über sensible Themen, die sie nur schwer mit ihren Eltern oder Lehrern besprechen können, wie sexuelle Gesundheit [58] oder sexuelle Identität [59].

Übersichtsarbeiten zur Wirksamkeit von *Social-Media*-Interventionen zur Förderung einer gesunden Ernährung berichten widersprüchliche Ergebnisse [13, 60]. Studien, die digitale Technologien und insbesondere soziale Medien als erfolgreiches Instrument für ernährungsfördernde Interventionen bewerten, unterstrichen ihr Potenzial, auch schwer erreichbare Gruppen zu erreichen [60]. Anderseits führte der Anblick von Influencer:innen, die gesunde Snacks zeigten, nicht zu einem höheren Verzehr gesunder Snacks [32].

Interventionsstudien zur Erhöhung der körperlichen Aktivität mittels digitaler Sportspiele, sogenannter Active Video Games oder Excergames, berichten bei Kindern und Jugendlichen entweder keine Erfolge [61] oder eine Verbesserung der körperlichen Aktivität und des Body-Mass-Index [62, 63]. Bei einigen Interventionen zur Förderung körperlicher Aktivität und zum gesunden Umgang mit digitalen Medien wird die Reduzierung der Bildschirmzeit als ein entscheidender Faktor für die langfristige Verbesserung der menschlichen Gesundheit gesehen [64]. Die Verringerung der Bildschirmzeit ging jedoch nicht immer mit einer deutlichen Zunahme der Aktivität einher [65], weil körperliche Aktivität bei Kindern und Jugendlichen kontextabhängig stattfindet, wie z. B. durch organisierten Sport, und sie trotz längerer Medienzeiten ausreichend aktiv sein können [66]. *Social-Media*-Interventionen zur Förderung von körperlicher Aktivität können zu einer Verbesserung der körperlichen Aktivität beitragen. Aber auch hier erschweren die rasche Entwicklung und die Vielgestalt von *Social-Media-*Plattformen die Erarbeitung klarer Empfehlungen [67].

Die Förderung der digitalen Gesundheitskompetenz kann das Gesundheitsverhalten und die Gesundheit von Kindern und Jugendlichen positiv beeinflussen und chronischen Erkrankungen vorbeugen. Geeignete Interventionen haben das Potenzial, die gesundheitsbezogenen Fähigkeiten und Verhaltensweisen von Kindern kurzfristig zu verbessern, benötigen aber längere Laufzeiten und wiederholte Messungen, um ihre Wirksamkeit abschließend bewerten zu können [68]. Aus Sicht der öffentlichen Gesundheit sind soziale und digitale Medien ein leicht zugängliches und wirkungsvolles – weil akzeptiertes – Instrument, um Jugendlichen gesunde Verhaltensweisen und Gesundheitsthemen zu vermitteln [69, 70]. Dennoch bleibt zu bedenken, dass Eltern und Familien in diesem Altersabschnitt bei der Überwachung der Nutzungszeiten und Inhalte eine zentrale Rolle spielen [69, 71]. Ferner ist die Autonomieunterstützung ein wesentlicher Bestandteil bei der Entwicklung der kindlichen und jugendlichen Selbstwirksamkeit [72]. Eltern können die Mediennutzung ihrer Kinder steuern, indem sie z. B. durch *Co-Surfen* ermitteln, welche sozialen Plattformen die Kinder besuchen, indem sie Regeln für die Nutzung digitaler und sozialer Medien aufstellen und deren Einhaltung überwachen [73].

## Politische und rechtliche Rahmenbedingungen

Unsere Ergebnisse legen nahe, dass die Exposition gegenüber bestimmten sozialen Medieninhalten negative Auswirkungen auf die Nahrungsmittelauswahl und das Essverhalten von Kindern und Jugendlichen haben kann, und stützen so den Appell, digitale Werbung für Lebensmittel und Getränke, die sich an Kinder und Jugendliche richtet, in diesen Medien stark einzuschränken [74]. Bislang haben nur wenige Länder verbindliche Regulierungen eingeführt, darunter Chile, Portugal und das Vereinigte Königreich [75]. Die Vorschriften gelten jedoch nur für Kinder bis zum Alter von 12 Jahren und lassen Jugendliche ungeschützt. In Deutschland stellte im Februar 2023 das Bundesministerium für Ernährung und Landwirtschaft (BMEL) einen Gesetzentwurf vor, der an Kinder gerichtete Werbung für Lebensmittel mit zu viel Zucker, Salz oder Fett künftig nicht mehr erlaubt; das Vorhaben ist im Koalitionsvertrag verankert [76].

Gründe für zu lange Medienzeiten können manipulative Designfunktionen sein, die in vielen digitalen Plattformen integriert sind, einschließlich Gaming- und Social-Media-Netzwerken. Technologische Designfunktionen haben zum Ziel, entweder die Medien‑/Spielzeiten zu verlängern oder die Nutzungshäufigkeit zu steigern, indem zur erneuten Beschäftigung mit der App, dem Spiel oder der Plattform aufgefordert wird [77, 78]. Die Medienkompetenz und die kognitiven Abwehrmechanismen junger Nutzer:innen sind aber noch nicht ausreichend entwickelt, um zu erkennen, dass das Verbleiben auf der Plattform, im Spiel oder in der App oder gepostete Inhalte nicht ihrem freien Willen oder den eigenen Gedanken entspringen [78]. Altersbeschränkungen zur Nutzung der sozialen Plattformen haben bislang wenig Wirkung und erhöhen die Sicherheit von Kindern im digitalen Umfeld nur begrenzt [78].

Die Digitalpolitik könnte junge Nutzer:innen ins Zentrum aller Bemühungen stellen, nicht nur, um sie vor gesundheitlichen Schäden zu bewahren, sondern auch, damit die Technologie ihnen hilft, ihr Potenzial voll auszuschöpfen. Daher braucht es verbindliche Regeln, um junge Nutzer:innen vor manipulativen Designfunktionen zu schützen. Auch wenn bislang die Inhalte und die Ausrichtung der digitalen Anwendungen vorrangig kommerziellen Zielen dienen, sollten Strategien darauf abzielen, die sozialen Medien in Zukunft gesundheitsförderlich und nachhaltig zu gestalten [79].

Für Deutschland beschreibt die Bundesregierung in ihrer Digitalstrategie, wie die Digitalisierung so gestaltet werden kann, dass alle Menschen von ihr profitieren können, unabhängig von z. B. Alter, Geschlecht oder sozialer Lage. Sie erklärt hierfür die 17 Nachhaltigkeitsziele der Vereinten Nationen als Richtmaß für die eigene Politik und explizit für die Digitalstrategie. Insbesondere das Nachhaltigkeitsziel Nr. 4 „Hochwertige Bildung“ möchte „inklusive, gleichberechtigte und hochwertige Bildung gewährleisten und Möglichkeiten lebenslangen Lernens für alle fördern“ [80].

## Limitationen

Zusammenhänge mit Gesundheitsendpunkten im Kindes- und Jugendalter können in dieser Arbeit nur für ein Medienverhalten untersucht werden, das zeitlich nicht mehr aktuell ist, denn Technologien entwickeln sich schnell und Studien können zumeist nur bereits überholte Inhalte und Funktionalitäten sozialer Medien untersuchen [60]. In Anbetracht der sich rasch verändernden digitalen Landschaft und der Komplexität des Themas werden vergleichbare Forschungsdaten benötigt, die durch standardisierte Protokolle in größeren Stichproben gemessen werden und Informationen über heutige Technologien und ihre Nutzung bereitstellen.

Die Datenlage erschwert also Schlussfolgerungen über kausale Zusammenhänge zwischen Medienzeiten, Inhalten sozialer Medien und Gesundheitsendpunkten bei Kindern und Jugendlichen. Der vorliegende narrative Übersichtsartikel soll den aktuellen Wissensstand zum Thema darstellen, ergänzt durch unsere eigenen Forschungsergebnisse, und erhebt keinen Anspruch auf Vollständigkeit.

## Fazit

Zusammenfassend zeigt sich, dass tägliche Medienzeiten, die 2 h überschreiten, das Risiko für Übergewicht, Adipositas oder das metabolische Syndrom von Kindern und Jugendlichen erhöhen. Eine tägliche Bildschirmzeit, die 5–6 h übersteigt, steht mit dem höchsten Risiko für die Entstehung des metabolischen Syndroms bei Kindern und Jugendlichen in Beziehung [20]. Die durch das digitale Umfeld begünstigten Veränderungen der Geschmackspräferenzen und des Ernährungsverhaltens junger Nutzer:innen scheinen diese Entwicklungen zu fördern.

Zur Erhaltung der metabolischen Gesundheit und des emotionalen Wohlbefindens junger Nutzer:innen sollte sich die Forschung auf Maßnahmen zur Verringerung der negativen Auswirkungen konzentrieren. Kinder und Jugendliche sind die Bevölkerungsgruppe, die am meisten von den digitalen Technologien profitieren kann und gleichzeitig am meisten gefährdet ist. Daher sollte zukünftige Forschung zu den gesundheitsfördernden Aspekten digitaler und sozialer Medien die Entwicklung kognitiver Fähigkeiten im jungen Alter berücksichtigen und junge Nutzer:innen bei der Entwicklung neuer Studiendesigns einbeziehen [57].
